# Hospital and Institutional News

**Published:** 1917-06-09

**Authors:** 


### HOSPITAL AND INSTITUTIONAL NEWS.

### BRITISH AMBULANCES WITH THE FRENCH ARMY.

A number of British ambulances have served with the French Army for many months, and some five of these have been much damaged by German shell fire, though it must not be assumed that the damage has always been intentional; but it is not improbable that intention has played some part in the injury. One of these damaged ambulances has been returned to England, and it will be on view at the foot of the Nelson Monument in Trafalgar Square until June 10. It is important that we should keep up to full strength our convoys attached to the armies of France, now that the great crisis of the war is upon us, and Lord Beresford is appealing for funds to enable the Ambulance Committee to replace these ambulances wrecked in so noble a cause. Contributions can be sent to the British Ambulance Committee, to the Service de Santé Militaire, 23a Bruton Street, W. 1.

### THE SCARCITY OF DOCTORS.

The constant demand for medical men to serve in the Army has naturally caused a very remarkable dearth of doctors in civil life, and this has clearly been shown by what has occurred at Manchester. The medical officer of health, Dr. Niven, has reported to the Corporation Health Committee that he has been unable to find a suitable applicant for the post of assistant medical officer at the Baguley Sanatorium. The post is worth £400 a year, and though apparently there have been some applications for the vacancy, not a single satisfactory application has been received. A salary of £400 a year is undoubtedly somewhat large, but it must not be forgotten that the pay of a lieutenant in the Royal Army Medical Corps is 24s. a day, amounting in the year to £438, though out of this he has to provide his messing money. Further, the recent action of the War Office in calling up for examination all medical men of military age, and practically insisting on their accepting commissions in the R.A.M.C., has made it almost impossible for a doctor under the age of forty-one to accept such a post as that at the Baguley Sanatorium unless he has some physical disability which unfits him for the rigours of war, though it is not unlikely that he would in such a case be also unfitted for the vacant post.

### THE FRUITS OF "THE HOSPITAL'S" TEACHING.

The Liverpool Council of Voluntary Aid has done good service in maintaining the issue of its Quarterly Paper, which is a file of the voluntary social work proceeding in Liverpool, and also of the chief Government publications which affect institutional affairs. The voluntary social worker in Liverpool is fortunate in possessing this pamphlet, which deals with such matters as the War Charities Act in operation, the number of voluntary appeals now before the public, and the like. It cannot be regarded as a newspaper, and is not intended as such, but as a local file it is of much value. It is now some thirty years since The Hospital was founded to preach the gospel of voluntary service, to record the developments of administrative medicine, and to reflect the activities of institutional life. The development of such bodies as the Liverpool Council of Voluntary Aid and the publication of its Quarterly Paper are gratifying proofs that the general public is awakening to the importance of the truths which The Hospital began to preach a generation ago.

### THE BARTLETTS AND EAST SUFFOLK HOSPITAL.

The death of Dr. J. H. Bartlett, of Ipswich, removes a member of a family which has many professional and other ties with the East Suffolk Hospital. On its establishment in 1836 the late Dr. Bartlett's father was the first member of the hospital's staff, and held the post for a very long period. The out-patient department, built in memory of Mrs. Bartlett, and the porter's lodge owed much to the late doctor, who was a constant and generous contributor to the hospital's funds. Dr. Bartlett, too, succeeded the late Mr. F. T. Cobbold as the hospital's president. The institution received from him an inscribed portrait of Lord Lister, who was his friend and fellow-student, and Dr. Bartlett is also remembered as the practitioner who performed a slight operation on Mr. Joseph Chamberlain when an attack of neuralgic toothache threatened to prevent him from delivering his so-called "ransom" speech at the Corn Exchange in 1885.

### A LAUGHABLE CONFESSION.

A movement has been started for raising a fund for the benefit of the numerous private hospitals for wounded officers in London. Convened by Mrs. Arnoldi, of the Roland Gardens Hospital, and patronised by Lord French and Sir Alfred Keogh, the fund is to have the benefits of a "Mascot Day" on July 17. The lure of the mascot was suggested by the explorer, Captain Besley, who has presented to the fund a small pre-Inca idol which was given to him by an old Indian woman, who said it would save him from a violent death. It is said to be 5,000 years old, and is valued by its late owner as having accompanied him in many emergencies, especially in a very narrow escape from death which occurred within a fortnight of the time that he received it. With amusing inconsistence the gallant captain, while disavowing any superstitious beliefs, declares that the continued possession of a mascot through many dangers steadies a man's nerve and gives him confidence to face them. So free is he from superstition, and so sure is his belief, that, though the public, after purchasing replicas of the original, is to be permitted to take part in a final draw for its possession, Captain Besley insists that the original shall be lent to him when the time for his next exploration comes round. A man's belief is to be discovered not from his professions, but from the assumptions on which he is accustomed to act, and superstition is only the name one gives to a belief one does not share. This story, with its ironic comedy, will give to each replica on sale on Mascot Day a certain *cachet* which the ordinary badge is wont to lack.

### PRESENTATION TO A DOCTOR.

A silver cup has been presented to Dr. J. Stirling-Hamilton for the work which he has done for the military hospital, Ingatestone, Essex. The inscription reads: "Presented to Dr. J. Stirling-Hamilton, M.B., B.C. (Cantab.), by Mrs. F. Hilder, Commandant, Essex, 102, in grateful appreciation of the valuable services rendered at the Huskards Auxiliary Military Hospital, Ingatestone, 1914/1917." The presentation was to have taken place on the occasion when the Countess of Warwick visited the hospital to present badges and certificates to the nurses, but owing to the doctor's absence, Mrs. Hilder undertook to present it to him herself.

### MRS. LLOYD GEORGE AT THE SWANSEA INFIRMARY.

The Swansea General and Eye Hospital Linen Guild—of which Lady Dynevor has been re-elected president—has afforded very valuable aid to the Swansea Hospital during the past year. The money worth of the Guild's gifts totalled £1,000, and now the hospital's nursing and domestic staff are to be provided with their uniform, at a cost of £120. That the Guild is run on sound financial lines may be inferred from the fact that its investments amount to £1,100. Mrs. Lloyd George, in the course of an address she delivered on a recent visit to Swansea Hospital, observed that even in peace times it was very difficult to get enough money to carry on a hospital, but in a time of war such as this the difficulty was much increased, so that often they were at a loss to know how to keep things going when subscriptions were wanted for so many objects. Commenting on what the matron, Miss Scovell, had said with regard to the need for further accommodation, Mrs. Lloyd George remarked that she hoped Swansea Hospital, as soon as the war was over, would be able not only to get the verandahs required, but to carry out its great scheme of extension.

### THE ALDERSHOT WORKERS' HOSPITAL FUND.

We are happy to note elsewhere the success which has attended the third year of the West Kent Hospital Pay Day Fund. Another satisfactory result on similar lines has attended the Aldershot District Workers' Hospital Fund, which has now reached a total of £451. Its first year realised £242, its second £332, so the progress has been steady and continuous. If this is still maintained, it is hoped to form the nucleus of a building fund, for Mr. T. W. Robertson, the hospital's president, foresees a time of great strain after the war, and believes that the after-effect of campaigns will breed a large amount of sickness with which the voluntary hospitals will have to cope. The immediate need, however, is for an installation of $x$-ray apparatus, which is estimated to cost £600, and for which adequate room has to be found. The Workers' Fund has direct representation on the committee, and this fact has helped to create a feeling of local solidarity which is and always must be a hospital's main support. An income which is not the expression of such a feeling is of no permanence; but with a solid body of active interest behind it any voluntary hospital is able to meet the demands of the future, and to expand its work as new needs arise.

### HOSPITAL AS A "MOBILISATION CENTRE."

We are informed that the War Office have decided to make an immediate but strictly limited trial of the scheme for organising hospital units suggested by Lieutenant-Colonel Sir James K. Fowler, K.C.V.O., to which we referred in our issue of May 26, p. 143. It has been determined that one of the additional units required may be raised from amongst the medical men educated at a single London hospital, who have not yet been able to serve abroad for various reasons or, having already done so, desire to renew their engagements for a further period. The Middlesex Hospital has been selected for the trial and Sir James Fowler has undertaken to form a "mobilisation centre" in connection with it. More than 400 Middlesex men are known to be now, or to have been some time during the war, serving as officers of the Royal Army Medical Corps, and other hospitals have an equally good record. The officers of this new unit will all be Middlesex men, the matron, sisters, and nurses will have been trained at that hospital, and the V.A.D.s will be selected from families connected with the hospital, but the orderlies will be men now serving with the Royal Army Medical Corps. The unit will be known as "The Middlesex No. —— General Hospital." No undertaking will be given that the *personnel* of this unit shall not be detached for service with other units, as may be found necessary. The unit will be raised for general service, and will be sent to any of the various theatres of war at which it may be required. It follows that the officers, nurses, etc., must all be fit for general service, and engagements will be for the duration of the war. No applications from Middlesex men now serving abroad for transfer to this unit will be considered except under special circumstances. Communications connected with this unit should in the first instance be addressed to Lieutenant-Colonel Sir James Fowler, or in the case of nurses and V.A.D.s to the Matron at the Middlesex Hospital, Mortimer Street, W.

### THE RED CROSS "VICTORY" SALE.

The announcement of the total proceeds of the great sale held at Christie's in March and April will come as something of a surprise to the most sanguine. That £84,000 should have been so collected is indeed a tribute to the seemingly fathomless charity of the British public. No one who was present will forget the atmosphere of generous outbidding which was so characteristic of the proceedings. Comparatively worthless works of art were sold for figures which must have made their artists turn in their graves, and the ubiquitous dealer kept well in the background, doubtless appalled at such wanton expenditure. This brings up the total of Red Cross contributions to the stupendous sum of £6,718,816. (Why the Society should allow the *Times* newspaper to claim the credit for the whole affair is not immediately apparent.) The proceeds of the sale are to be devoted to the interests of British prisoners of war in enemy countries, an object which, if current reports are to be believed, needs all the support it can get. Certainly it has fully justified the hope expressed by Mr. Hannen, one of the partners of the famous art firm, on the opening day, that it would be a "victory sale."

### RED CROSS WORK IN SUSSEX.

THE Mid-Sussex Division of the British Red Cross Society held its annual meeting on Tuesday, when the Countess of Chichester presided. The meeting was held at The Shelleys, Lewes, a house well known in the town into which the hospital moved at the beginning of the year. The house, some of which is very old, has certain associations, which include a visit from Dr. Johnson, who is said to have been so worried by a little child's questions as to have rid himself of her importunity by placing her in a tree, when he forgot all about her until a hue and cry was raised, and then he explained what had occurred. Like all the houses in Lewes which lie on the north side of the High Street, The Shelleys only gets the sun upon its front, most of which is occupied by a big hall. The big rooms, which include later additions, are all at the back, and therefore it is not as suitable for conversion as its apparent size and local repute might lead a casual observer to suppose. The income for the year 1916 amounted to £733 and the expenditure to £638. The number of beds in the various hospitals of the division shows an increase, and the *personnel* has correspondingly enlarged.

### THE LORD MAYOR AT DOLLIS HILL.

ST ANDREW'S HOSPITAL, Dollis Hill, in spite of its ups and downs, manages to keep in the public eye, and lately celebrated its Founder's Day by a visit from the Lord Mayor, who handed over a sum of £1,600 as the first fruits of the appeal which he is issuing to pay off the building debt of £9,000. Sir William Dunn, who arrived in civic state, was thanked by Mr. Edward Eyre and Cardinal Bourne, and the latter paid a tribute to the work of the sisters of mercy in nursing the wounded.

### ANOTHER BUILDING FUND.

THE Birmingham and Midland Hospital for Nervous Diseases, which has been eager for some time to extend the scope of its work by the provision of beds for in-patients, has started a building fund, to be drawn upon as soon as opportunity occurs. At present a sum of £1,300 has been obtained, and if the interest in the proposed scheme can be upheld there is no reason why it should not steadily advance towards the required total. It is not unlikely that the difficult time for raising money will occur immediately after the conclusion of peace, when business competition, demobilisation, attempts to get back to the pre-war output, and increased taxation may make the public more slow in giving than they are to-day, when wages are high and employment easy. The promoters of building funds, therefore, may look back on the present time as an occasion of which advantage was or was not taken, and we advise all with such schemes in hand to make the most of it.

### HOSPITAL EXTENSION AT NOTTINGHAM.

THE Bayley Auxiliary Hospital, Derby Road, Nottingham, has received an important extension by the opening of the Parkhill Congregational Sunday School, which will allow a further thirty beds for soldiers to be provided. The new building, which was opened by the Duchess of Newcastle, is intended to be used for convalescent soldiers, and has been equipped at a cost of about £500. Miss L. Birkin has held the post of matron from the beginning, and Mrs. Huskinson is the acting quartermaster.

### THE WEST KENT HOSPITAL PAY DAY FUND

WORKERS' collections and shop funds have been of somewhat slower growth in the South of England than in the Midland cities and the industrial districts further north. Cambridge, however, made the attempt some years ago, and its District Workers' Fund has been successful. Another good example is the West Kent Hospital Pay Day Fund, which was established three years ago for the benefit of the West Kent Hospital. Originally, forty-three firms contributed £112. In the second year the number had risen to fifty-eight, and the total to £456. Last year ninety-five firms contributed, and the total sum amounted to £661. This is a very encouraging record, which we hope may lead to the establishment of similar funds in other county towns. Mr. Gerald C. Mercer, the honorary secretary, has been very energetic on its behalf, and if his aim of raising £1,000 by the fund is soon realised, the hospital will largely have to thank him for it.

### A WAR COUNCIL CONCESSION.

THANKS to the action taken by the Southwark Board of Guardians, through the representatives of the borough in the House of Commons, the War Office have now decided to grant charge-pay to commissioned officers in charge of military hospitals established in Poor-Law institutions. The War Office some weeks ago stopped the charge-pay to these officers, with the result that they were seriously affected financially. This pay was permitted in the case of war hospitals entirely equipped and maintained by the Army Council, and it was obvious that the heavy responsibility resting on the officer in charge for the administration of a large hospital, such as that of Southwark, equally entitled him to the same financial assistance. In the case of Major Bruce, at the Southwark Military Hospital, the War Office have decided to issue his charge-pay from the date of its suspension.

### A MOTOR LABORATORY FOR THE FRENCH RED CROSS.

A motor bacteriological laboratory has been built for the use of the French Army, and it has been provided at the cost of the London Committee of the French Red Cross. It was designed by Dr. Tilmant, of the French Army, in conjunction with Dr. Garstang, of Liverpool. It is extremely compact; and by means of extensions it can be enlarged so as to form a room about 18 feet by 12 feet. The scientific fittings have been provided by Mr. Robert Mond, of Combe Bank, Sevenoaks, the brother of Sir A. Mond. Electric light is provided in the laboratory; it contains all the latest bacteriological appliances, and it is capable of the best bacteriological work. Everything is so arranged that when required to go off to another station the whole can be packed up in a very short space of time. The total cost of the motor laboratory and its fittings is about £1,500. A somewhat smaller motor laboratory has been constructed by the order and at the cost of the British Red Cross, for the purpose of presentation to the French Red Cross.

### THE EFFECTIVE USE OF RADIUM.

At the Health Committee of the Hull Corporation, the medical officer of health submitted details of his suggestions for the conversion of several of the radium applicators into one applicator, so that the radium might be more effectively used, and also as to other minor alterations in other applicators. The Town Clerk reported that he had communicated with Mr. Fierke, the secretary of the Voluntary Radium Committee, as to whether there was any objection on the part of that committee to the alterations suggested, and from the reply it appeared that Mr. Fierke had not been able to call the committee together, and that his view was that the matter had better be left with the Corporation, which must be guided in its course by expert advice. The Health Committee has decided to adopt the recommendation of the medical officer of health, subject to his consulting Dr. Holder on the matter.

### A SOLDIER'S TRIBUTE TO HOMŒOPATHY.

In response to the appeal, already noted in The Hospital, for a sum of £20,000 to acquire the site and equip the new Homœopathic Hospital at Redland, Bristol, which Mr. W. Melville Wills generously offered to build, the following letter has been received from a wounded soldier:—"Please accept my grateful thanks for your detailed reply to my inquiry concerning the proposed new hospital scheme. Your circular speaks of the need of small contributions as well as large ones. As one who has received benefit from homœopathic treatment, I shall be glad to send you six penny stamps weekly from my very limited income as a soldier, and by separate post you will receive a gold ring, thin and worn, perhaps, but none the less valuable to me, which I should like you to realise for the fund. This ring is very precious to me on account of its associations, but that is why I send it. I feel that one could not give too much to the object you have in view. . . If there is anything further that a soldier could do to show his gratitude, please let me know." The response to the appeal has made a good start, and if the whole sum is raised, the new building at Redland Green will be used for in-patients, while the building at Brunswick Square will be relegated to out-patients only.

### IS A HEALTH VISITOR A LUXURY?

After an animated discussion the Chelmsford Town Council has decided not to proceed with the appointment of a health visitor so long as the demand for qualified nurses for war work continues. This determination, which was made after the President of the Local Government Board had declined to concur in the postponement, was upheld by the Deputy-Mayor, who declared that Chelmsford was not much larger than a village, and further, that everyone who was fit and capable of being a nurse could do more good at the Front than if she remained at home. The Mayor reminded the meeting that the medical officer was anxious that the appointment should be made, and since nurses cannot be supplied to the Front by such sporadic private enterprise at home as the Deputy-Mayor appears to favour, the Town Council's decision is to be regretted. A health visitor at home is quite as important as a nurse abroad, and the notion that the only way to supply the needs of the Front is to dock every useful occupation at home is one which will not stand the strain of five minutes serious attention.

### THIS WEEK'S DRUG MARKET.

Business has again been quiet, although slightly better than last week; price changes of importance have been few. Salicylates are still in limited supply, and in active demand at dearer rates; one of the reasons for the shortage of supply is that the increased output from British factories enabled the drugs to be sold at prices with which American makers were not over-anxious to compete; but now that American supplies have been so considerably reduced, the output from British factories is insufficient to meet the full demand. Cocaine is less freely offered. Lithia carbonate and lithia citrate are slightly dearer. Makers of strychnine have again advanced their quotations. Podophyllin is dearer. Methyl salicylate (artificial oil of wintergreen) tends upwards in price in sympathy with other salicylates. Extreme prices are still asked for barbitone of good quality, but as it is reported that there are parcels of somewhat uncertain quality on the market, buyers should act with caution.

### TO OUR READERS.

**Contributions are specially invited from any of our readers to these columns. They should deal with topical subjects and news. They must be authenticated for the information of the Editor only. The minimum payment if published is 5s. There is no hard-and-fast rule as to space, but notes of about twenty lines in length are preferred.**

